# Strategies for Clarifying Penicillin Allergies When Skin Testing Is Not an Option

**DOI:** 10.3390/pharmacy7020069

**Published:** 2019-06-19

**Authors:** Elizabeth W. Covington, Mary Joyce B. Wingler, Rebecca A. Jayakumar, C. Whitney White

**Affiliations:** 1 McWhorter School of Pharmacy, Samford University, Birmingham, AL 35209, USA; 2 Antimicrobial Stewardship Program, University of Mississippi Medical Center, Jackson, MS 39216, USA; mwingler@umc.edu; 3 Pharmacy Practice, Roseman University of Health Sciences College of Pharmacy, Henderson, NV 89014, USA; rjayakumar@roseman.edu; 4 School of Pharmacy, University of Mississippi, Jackson, MS 39216, USA; cwhite9@umc.edu

**Keywords:** allergy, antibiotic, penicillin, beta-lactam, skin testing

## Abstract

Patients with reported penicillin allergies have been proven to experience negative health consequences, such as increased cost, suboptimal antimicrobial therapy, and adverse reactions. Though skin testing has been proposed as a method to clarify penicillin allergies, many institutions may lack the resources to perform skin testing on a wide scale. This literature review describes the current literature surrounding the use of penicillin allergy interviews when skin testing is not an option. Specifically, the review highlights the steps in carrying out a successful antibiotic allergy patient interview, summarizes the clinical evidence surrounding antibiotic allergy clarifications, and addresses key advantages and disadvantages of clarifying antibiotic allergies without the availability of skin testing.

## 1. Introduction

Approximately 25% of patients report having at least one allergy to an antimicrobial, and a penicillin allergy is the most commonly reported drug allergy, with a prevalence of around ten percent [1,2]. Yet among patients with reported penicillin allergies, approximately 90% do not have a positive penicillin skin test, demonstrating lack of detectable penicillin-specific IgE-antibodies [3,4]. Furthermore, up to 80% of patients with a history of a true penicillin allergy lose sensitivity to penicillin after a period of 10 years [5]. Data have shown that over 50% of “allergies” are non-immunologic and represent adverse drug reactions or intolerances, such as nausea [6]. Additionally, self-reported drug allergies have limitations, including details of the allergic event being absent or lacking appropriate descriptors in the medical record and drug intolerances or side effects inaccurately labeled as an allergy [7,8,9,10,11,12,13,14]. Inaccurate allergy documentation, specifically related to antimicrobials, increases the use of potentially less effective second-line agents, increases health care costs and hospital length of stay, and leads to higher rates of resistant organisms, such as methicillin-resistant *Staphylococcus aureus* (MRSA), *Clostridioides difficile*, and vancomycin-resistant enterococcus (VRE) [2,6,8,15,16]. Due to the impact of inaccurate drug allergy reporting and documentation on patient care, the Infectious Disease Society of America (IDSA) recently published guidelines, which included effective allergy assessments as part of antimicrobial stewardship programs [17].

Penicillin skin testing has been described and utilized as an effective tool for identifying true penicillin allergies but widespread availability and implementation of skin testing is lacking. When available, interpreting the results of penicillin skin tests (PST) should be done with caution. Studies have shown PST to have a high negative predictive value, though lower than originally thought, and PST’s have a relatively low positive predictive value (~50%) with approximately 10% of patients having a positive test. Of note, a patient with a negative skin test may still be allergic to a penicillin, though unlikely via an IgE-mediated reaction. Additionally, if skin tests are negative, an oral controlled challenge may be warranted [18,19]. Thus, the clinical use of PST can be limited, despite access to the testing.

As an alternative to PST, in-depth patient interview can provide meaningful information regarding incidence and severity of drug allergies and avoid unnecessary therapeutic substitutions. The interview to clarify drug allergies can provide information related to type or severity of reaction to a medication, differentiate drug intolerances, sensitivities, or expected drug effects documented as a drug allergy, and identify documented drug allergies in the medical record that the patient does not endorse on interview [20]. Studies have shown that discrepancies between allergy documentation in the medical record and patient interview are common [20,21] and that 30 to 63% of patients can be “de-labeled” after interview and review of the medical record and medication history [12,22]. This review will describe the key parts of a successful patient interview, summarize published evidence on the impact of antibiotic allergy clarifications, and discuss the advantages and disadvantages of allergy clarification in the absence of skin testing. 

## 2. Review of the Literature

### 2.1. Conducting Antibiotic Allergy Clarifications

A comprehensive allergy history is a key component to a successful antibiotic allergy evaluation. There are currently few validated allergy history questionnaires or risk assessment scores available. To determine “best practices” for performing antibiotic allergy clarifications, a literature search on PubMed was performed using search terms “antibiotic”, “beta lactam,” “penicillin,” “allergy,” “hypersensitivity,” “reaction,” and “immune mediated hypersensitivity,” along with the following terms: “interview,” “questionnaire”, “clarification”, “delabel,” “evaluation.” Allergy interview questions described in prior literature were noted, grouped by content/theme, and summarized in Table 1 below. Case reports and animal studies were excluded from the review. 

Current literature cites numerous health care professionals involved in antibiotic allergy clarifications, including pharmacists, nurses, physicians, medical residents, infectious disease specialists, antimicrobial stewards, and allergy specialists [23]. It may be reasonable to train those participating in the medication reconciliation process to obtain a thorough allergy history while interviewing the patient about their medication history. Pharmacists may be uniquely posed to conduct allergy interviews due to training about antibiotic classes and cross-reactivity. In fact, a study by Nester et al. found that pharmacists identified more discrepancies regarding medication allergies than nurses [24]. The study also found that pharmacist-led allergy clarifications resulted in faster entry of allergy information into the chart by 88 minutes (156 min vs. 68 min, p < 0.005) [24]. This could have important clinical implications for infections in which administration time of antimicrobials has been associated with improved patient outcomes, such as meningitis and sepsis [25,26]. Similarly, a study by Devchand et al. showed improved performance of an allergy assessment tool when carried out by pharmacists and senior doctors [27]. A recent study by Covington et al. described the use of fourth-year pharmacy students as pharmacist extenders to clarify beta-lactam allergies, with supervision from clinical pharmacists and/or pharmacy residents [28]. This could represent a strategy for institutions with advanced pharmacy practice experience (APPE) students. 

Recommended questions to ask a patient when performing an allergy interview are listed in Table 1. These questions can characterize the patient’s reaction (or lack thereof), determine likelihood of causality, classify the reaction as IgE-mediated or non-IgE mediated, verify time frame, and uncover patient history with other antimicrobials. Rationale for questions included in the interview are listed below.

**Characterization of the reaction:** Obtaining a thorough allergy history can provide information to help distinguish between a true allergy and non-allergic adverse reaction (NAAR). In a cross-sectional study of 426 hospitalized patients by Torda et al, 25.6% of patients described NAAR [29]. Comprehensive allergy histories are also crucial in determining the severity of a patient’s antibiotic allergies and potential risk/benefit of administering a similar class of antibiotic. Immediate urticarial, angioedema, and/or anaphylaxis within 24 h are indicative of an IgE-mediated allergic reaction, for instance [30]. Non-IgE mediated reactions may present as delayed maculopapular rash, or in severe cases, Stevens Johnsons Syndrome (SJS) or toxic epidermal necrolysis (TEN) [23]. Gastrointestinal symptoms or headache may indicate a NAAR. Of note, however, immediate gastrointestinal symptoms may occur in patients with IgE-mediated acute anaphylaxis [31]. By performing an allergy clarification, it may even be determined that a patient has never experienced symptoms of an allergy or NAAR. In the above-mentioned study, 7% of patients denied any allergy and claimed to tolerate the antibiotic [29]. 

**Likelihood of causality:** Allergy histories can also provide insight into the likelihood that a patient’s reaction was caused by the antibiotic versus another medication or perhaps an underlying illness. For example, if a patient says that penicillin was prescribed for streptococcal pharyngitis, a history of rash may have been due to the infection itself, rather than the penicillin. Within the cross-sectional study by Torda et al, 2% of patients were classified as having reactions likely as a result of the disease at the time [29]. Several validated adverse drug reaction scales exist that assist with the causality determination. Such scales include the Naranjo adverse drug reaction (ADR) probability scale, the Liverpool ADR causality assessment tool, ALDEN, among others [32]. However, causality tools alone may not provide enough information to sufficiently clarify a penicillin allergy without additional questions regarding reaction characteristics, timeframe, history of allergy testing, etc., as mentioned throughout this text. In fact, a recent study showed poor reliability and validity of ALDEN, Naranjo, and Liverpool scoring tools to identify drug-induced SJS and TEN [32]. This emphasizes the importance of a comprehensive allergy questionnaire to appropriately identify and clarify antibiotic allergies. 

**IgE-mediated versus non-IgE mediated**: Additionally, patient interview can help determine if a patient’s allergic reaction was IgE-mediated versus non-IgE mediated. Typically, IgE mediated reactions occur within minutes to hours after the last drug administration. Clinical presentations of IgE-mediated reactions commonly include itching, wheezing, urticaria, anaphylaxis, or angioedema. Non-IgE mediated reactions include IgG, IgM, and T cell-mediated reactions. Non-IgE mediated reactions typically manifest days to weeks after drug exposure, in contrast with IgE reactions [23,33]. Clinical presentations of IgG mediated reactions include hemolytic anemia or vasculitis. IgM reactions include serum sickness, fever, rash, or arthralgias. T cell-mediated reactions are typically heterogeneous in nature, but may include delayed rash, liver injury, drug reaction eosinophilia and systemic symptoms syndrome (DRESS), SJS, or TEN [33]. 

**Timeframe of the reaction**: If an allergy interview does reveal a likely IgE-mediated allergy, it is also important to determine how long ago the reaction occurred. A recent retrospective study by Siew, et al. determined that a reaction occurring more than 1 year before testing, combined with an absence of anaphylactic severity and a patient’s inability to recall the name of the index drug, has a negative predictive value (NPV) of 98.4% for type I beta-lactam allergy [34]. Studies have shown that IgE antibiotics can decline and disappear over time. In fact, 50% of patients with IgE-mediated reactions lose sensitivity within 5 years, and 80% lose sensitivity within 10 years [35]. However, it is important to note that this phenomenon is limited to penicillin-specific IgE antibodies, and does apply to non-IgE mediated reactions. Additionally, reduction in penicillin-specific IgE antibiotics does not occur for all patients [36]. 

Because allergic reactions require previous exposure to launch an immune response, it is also important to determine if a patient experienced a reaction after the very first exposure to an antibiotic, or after a re-exposure [23]. A reaction to the first antibiotic exposure would likely represent a non-immune mediated reaction or a rare cross-reactivity to an unrelated drug class. 

**Receipt of other antibiotics**: Determining a patient’s experiences with other antibiotics is a key piece of the allergy clarification process. Oftentimes, patients with labeled penicillin allergies may claim to have tolerated specific penicillins with no reaction. For example, a patient may claim to have tolerated amoxicillin/clavulanate in their lifetime, suggesting that his or her penicillin allergy label may be inaccurate. Patients may also report tolerating other beta-lactams, such as a cephalosporin or carbapenem, which also provides pertinent information ruling out cross-reactivity. Furthermore, a thorough allergy clarification may confirm the identity of the culprit drug, as a patient may have mistaken the name of the offending medication.

**History of skin testing and other drug allergy testing**: Patients may have already seen an allergy specialist and/or had penicillin skin-testing performed. This information should not be forgotten, as penicillin skin testing remains the most reliable method for identifying IgE-mediated allergic reactions [3]. Other tests that patients may have had performed include the basophil activation test (BAT), enzyme-linked immunosorbent spot (ELISpot), lymphocyte transformation test (LTT), and immunoassays to detect drug-specific IgE [37]. Details from these tests can provide valuable information to the health care team regarding type and details of the patient’s reaction.

**After interview completion**: Some patients may remain unclassifiable after interview alone. In a study by Torda et al, 9% of patients remained unclassifiable after allergy interview [29]. In such cases, there are other strategies that can be employed to further clarify a patient’s antibiotic allergy. First, the patient’s medication profile can be reviewed for receipt of antibiotics during the current admission or during previous admissions to the institutions. Receipt of antibiotics in the emergency department should also be reviewed. Second, outpatient pharmacy claims data can be reviewed, if available, to assess a patient’s history of outpatient antibiotic prescriptions. Third, consider calling the patient’s community pharmacy for allergy details, as well as antibiotic fill history. After completing these additional steps, some patients’ antibiotic allergies will remain elusive. If such patients require beta-lactam therapy for their current infection, an oral graded challenge, desensitization, and/or penicillin skin testing may be considered.

The decision to perform additional test(s) after an allergy clarification interview should be based on several factors, including type and timing of reaction [31]. Oral challenges can typically be performed without the use of skin testing in patients with low-risk penicillin allergy histories. Low-risk typically encompasses patients who report an unknown reaction occurring >10 years ago, non-allergic symptoms, or pruritis without rash [31,38,39]. Despite taking precautions to select patients in whom an allergic reaction is unlikely to occur, the risk still exists; therefore, oral challenges should be performed by a trained specialist in a setting where close monitoring can occur. In an oral challenge the drug, most commonly amoxicillin, is administered either as a single dose, in divided doses (graded challenge) or multiple doses (prolonged challenge) [31,38,39]. The results of the oral challenge should be evaluated by the specialist.

After successfully completing an antibiotic allergy clarification and other tests, it is important to educate the patient about their allergy details. For instance, if a patient is deemed to have experienced a non-allergic adverse reaction (NAAR) or is found to have tolerated a similar antibiotic in the past, it is important to explain this to the patient lest the antibiotic allergy label persist when giving an allergy history at future health care encounters. On the other hand, patients reporting previous reactions who are deemed eligible for further testing should have a thorough discussion about risks, benefits, and options. The medical record should also be updated with information obtained from the allergy interview and further tests, if performed, to assist all health care professionals at the institution when making clinical decisions about the patient’s care.

**Table 1:** Antibiotic allergy interview questions.

**Characterization of the Reaction**
*Describe the reaction that you experienced.* *What were the signs and symptoms?*
**Likelihood of Causality**
*Were you taking other new medications at the time of the reaction?* *What happened after you stopped the antibiotic?* *Have symptoms similar to the reaction ever happened to you in the absence of medication therapy?* *Why was the antibiotic prescribed?*
**IgE- vs. Non-IgE Mediated Reaction**
*How many doses had you taken before the reaction?* ☐ 1☐ 2–3 ☐ 3–6☐ >6 *Did you experience throat closing, difficulty breathing, tongue/lip/facial swelling?* *Did you require medical treatment for the reaction (antihistamines, epinephrine, hospitalization, etc.)?*
**Timeframe of the Reaction**
*How many years ago did the reaction first occur?*☐ <1 ☐ 1 to <5 ☐ 5 to <10 ☐ 10 or more *Has the same medication been used since the previous reaction?*
**Receipt of other Antibiotics**
*What antibiotics do you normally take when you are sick?* *If patient unsure, list names of common antibiotics in your area (brand and generic).* Example: amoxicillin (Amoxil^®^), amoxicillin/clavulanate (Augmentin^®^), cephalexin (Keflex^®^), cefdinir (Omnicef^®^), cefixime (Suprax^®^)
**History of Skin Testing and Other Drug Allergy Testing**
*Were you referred to an allergy specialist?* *Have you had a penicillin skin test performed, or oral challenge? If so, what were the results?*

Interview questions adapted from [22,23,28,29,30,40,41,42,43].

### 2.2. Impact of Antibiotic Allergy Clarifications on Clinical Outcomes

Although clarification of allergies is a logical step to improving patient care, there are limited data on the clinical and economic impact of clarifying antibiotic allergies. Briefly summarized below is a group of diverse studies which have evaluated the impact of clarifying beta-lactam allergies.

In a pharmacist-driven pilot study over a 6-month period, 32 patients were interviewed to clarify beta-lactam allergies. Of the 24 patients who were eligible for a beta-lactam antibiotic, 21 patients (87.5%) were switched to a beta-lactam antibiotic and therapy was narrowed for an additional five patients who were already receiving either a third-generation cephalosporin or a penicillin. None of the patients who received a beta-lactam experienced a hypersensitivity reaction during their hospitalization. Of note, a discrepancy between the electronic medical record (EMR) and history obtained during the pharmacist interview was identified in 11 patients (34.4%), emphasizing the impact of the pharmacist interview on clarifying reactions of the beta-lactam allergies [22]. 

In a quasi-experimental study with a prospective cohort and a historical control, patients who reported a beta-lactam allergy and received levofloxacin as a beta-lactam alternative were included for a standardized beta-lactam allergy interview. No statistical differences at baseline were noted between the control group (n = 43) and the prospective group (n = 37). The mean duration of levofloxacin administration was statistically lower in the prospective group compared with the control group (2.7 days vs. 3.7 days, respectively, p = 0.026). Within the prospective group, 18 patients (49%) were switched to a beta-lactam after the interview and no adverse reactions were noted. Interestingly, 41% of those interviewed reported receiving a beta-lactam antibiotic in the past with no reaction which again emphasizes the importance of conducting a thorough allergy interview [28]. 

In a prospective, interventional study, pharmacists interviewed 250 patients with a reported penicillin allergy and followed records for one year after the intervention. Eighty percent of patients (n = 199) were found to have an incorrect penicillin label and had their allergy labels removed. Changes to antibiotic therapy were recommended in 61% (91 of 149 patients) of the patients with their allergy de-labeled and none of these patients experienced adverse events. Patients who had their allergy inaccurately labeled initially had a shorter duration of stay of six days compared to nine days for those who were confirmed allergic (p = 0.0015). Furthermore, the antibiotic cost for confirmed allergic patients was 1.6 times greater in the inpatient setting and 2.5 times greater in the outpatient setting compared with patients who had their allergy de-labeled [40]. 

A study evaluating the impact of a standardized beta-lactam allergy questionnaire on aztreonam utilization as an alternative to beta-lactam therapy included 95 patients in the pre-implementation and 65 patients in the post-implementation group. The average number of aztreonam doses per 1000 patients and average days of therapy was lower in the post-implementation group compared with the pre-implementation group (21.23 vs. 9.05, p = 0.003 and 8.79 vs. 4.24, p = 0.016, respectively). Similarly, there was an increase in the number of aztreonam de-escalations (65% vs. 85%, p = 0.003) and therapy changes to a beta-lactam (19% vs. 50%) post-implementation. There was no adverse events or allergic reactions noted in either group. In this four-month post-implementation period, a $12,889 cost savings was observed with an estimated annual savings of $37,857 [41]. 

In a historically controlled, quasi-experimental study, a myriad of interventions including medical staff education, aztreonam restriction protocols, and prospective interventions with prescribing clinicians were undertaken to reduce aztreonam utilization. A total of 211 patients were reviewed with 90 patients included in the control group and 63 patients included in the intervention or prospective group. A statistically significant reduction in median days of aztreonam therapy (4.0 vs. 2.0, p = 0.0001) and days of therapy per 1000 patient days (14.5 vs. 9.3, p = 0.0001) was observed. No differences in hospital length of stay or in-hospital mortality were identified. Furthermore, 52 of the intervention patients (83%) underwent antibiotic modifications with 31% (n = 16) discontinuing aztreonam and 69% (n = 36) changing to beta-lactam with superior coverage. None of these patients experienced an allergic reaction [44]. 

A retrospective study evaluating the impact of an interdisciplinary antimicrobial stewardship program on 186 patients with a self-reported beta-lactam allergy who were prescribed aztreonam. The median time to discontinuation of aztreonam was shorter after implementation of the antimicrobial stewardship program than prior to implementation (12.7 vs. 30.7 h, p = 0.02) with a reduced median aztreonam cost from $1285 to $402 (p = 0.011). After implementation of the antimicrobial stewardship programs, the number of patients switched to a beta-lactam antibiotic increased (34.6% vs. 22.8%) and no adverse effect observed in any of the study participants switched. Furthermore, there was no difference in rate of clinical cure (91.6% vs. 93.3%) between the post- and pre-antimicrobial stewardship program, respectively [45].

In a single center, retrospective, observational study, 280 patients with a self-reported penicillin allergy who received antibiotics with gram-negative coverage for at least 48 h were evaluated before and after implementation of a penicillin allergy education initiative. Aztreonam and fluoroquinolone usage were monitoring as alternatives to beta-lactam therapy in penicillin allergic patients. The clinical response rate improved from 83.6% to 91.4% in the post-implementation group (p = 0.0468). Additionally, the study found more cephalosporin use (p < 0.001) and less aztreonam (p = 0.017) and fluoroquinolone (p = 0008) usage in the post-implementation group compared with the pre-implementation group. Despite improved clinical outcomes, no differences in secondary outcomes including length of stay, survival rate, intensive care unit admission, and *Clostridioides difficile* infections were identified; however, the pre-implementation group had a higher rate of discontinuation of the antibiotic due to adverse reactions (4.3% vs. 0%, p = 0.03). The majority of adverse reactions were rash and swelling in those receiving a fluoroquinolone [46]. 

Based on the literature search conducted, the studies by Estep, et al. and Phan, et al. are the only two that evaluated the clinical outcome of clarifying beta-lactam allergies [45,46]. Though more studies are needed to confirm the impact of allergy clarifications on patient outcomes, these studies suggest an avoidance of adverse reactions from broad-spectrum antimicrobials, and perhaps an improved clinical response rate. These clinical outcomes coupled with improved allergy reaction documentation, reduced alternative antibiotic utilization and the safe administration of beta-lactams in patients with clarified allergies seem to emphasize the importance of clarifying antibiotic allergies.

### 2.3. Advantages/Disadvantages of Interview-Based Allergy Clarifications

As described above, interview-based clarification of penicillin allergies can be an excellent tool for evaluating the severity and time course of an allergy and for risk stratification of patients. However, there are many advantages and disadvantages to consider when utilizing this strategy alone.

The primary disadvantage to interview-based clarification is the inability to definitively rule in or out a drug allergy. Though many penicillin allergies wane over time, a significant proportion of patients cannot be de-labeled if further testing is not performed [31,35]. Risk stratification systems can help by identifying low risk patients in whom an allergic reaction is unlikely; however, the patient’s ultimate response will be unknown until a drug challenge and/or penicillin skin testing is completed, and most risk stratification tools remain unvalidated [31]. An additional limitation is the fact that allergy histories are often incomplete or inaccurate [18,23]. Many patients are unable to identify the drug, reaction, time course, and other important details related to their reported allergy. A review of 30 studies evaluated patients with positive penicillin skin tests, revealing 33% (range 0% to 70%) had a vague penicillin allergy history [47]. Another disadvantage is that interpretation of penicillin allergy histories is widely varied, and providers without proper training may be unaware of best practices [48,49]. A recent survey at two community teaching hospitals demonstrated heterogeneous management of patients with penicillin allergies, understanding of cross-reactivity with non-penicillin beta-lactams, and possibility of penicillin allergies resolving over time [48]. Another survey conducted at a single center showed 99% of health care staff understood the problematic nature of incorrectly documented penicillin allergies, though respondents cited barriers to clarifying patient allergies including time, lacking the knowledge to explain allergies and reactions, not feeling it was that individual’s role, and patients not accepting of their explanation [49]. These studies demonstrate that education is needed in order for medical staff to respond appropriately to a penicillin allergy history. Lastly, patients may be fearful of taking a beta lactam antibiotic based on a history-based allergy clarification, without objective testing [50]. 

While there are limitations to interview-based clarification, there are several advantages to consider. One significant advantage is the flexibility of personnel who can be involved in obtaining a penicillin allergy history. Education and training can be provided to a wide variety of health care providers, including nurses, pharmacists, physicians, and even trainees, such as medical or pharmacy students, residents, or fellows [22,27,41,51,52]. Another advantage is cost savings. One means by which costs can be decreased is identification of patients who do not require further testing for de-labeling. A patient interview can establish patients with non-specific, non-immunological reactions, such as headache or gastrointestinal upset, and de-labeling can occur without the need for penicillin skin testing or oral challenge [29,50]. Studies have also shown a cost benefit when utilizing preferred beta-lactams compared with alternative antibiotic classes or aztreonam [6,41,51,53,54,55,56,57]. For example, use of vancomycin requires therapeutic drug monitoring, which can involve a significant amount of time and resources. Another important consideration is the time involved in interview-based clarification compared with alternatives methods. Health care providers in all settings (outpatient, inpatient, nursing facilities, etc.) are increasingly pressured to provide additional services and value, often without an increase in resources. All other interventions, including drug challenge, skin testing, and desensitization, require considerable amounts of time for training, preparation, and administration of the drug or test [31]. Furthermore, it is recommended that individuals with specialized training perform these additional tests [31]. For non-urgent situations, this commonly requires an outpatient referral, which can cause delays in de-labeling and may lead to utilization of alternative antibiotics for the current infection. Outpatient referrals also may not be available in all areas. Skin testing has additional limitations, including a poor correlation with oral challenges and clinical outcomes and variability of test execution and result interpretation [58].

Apart from the advantages regarding resource utilization, a detailed history can allow for the use of alternative beta-lactams in patient who retain the penicillin allergy label, which can still provide improved patient outcomes [59]. For example, Blumenthal et al. evaluated the use of three treatment strategies in patients reporting penicillin allergies who had methicillin-sensitive *Staphylococcus aureus* bacteremia: (1) patients did not undergo an allergy evaluation and received vancomycin; (2) patients underwent an allergy history alone; (3) patients had an allergy history performed with a history-appropriate penicillin skin testing [59]. In the allergy history-guided treatment approach, patients with no reported anaphylactic features with penicillins were prescribed cefazolin. Clinical outcomes were better in both groups with an allergy evaluation compared with the vancomycin group, including incidence of adverse drug reactions. Furthermore, the group of patients who underwent skin testing only had marginally better clinical outcomes than those with an allergy history alone [59].

## 3. Discussion

An effective antibiotic allergy evaluation begins with a comprehensive allergy history. Though no validated questionnaire or risk assessment tool is available, several published studies evaluate the use of different methods and questions, which are summarized in Table 1. Fortunately, the detailed allergy interview can be conducted by a number of health care professionals, yet proper education and training remains a barrier to widespread use and application of best practices. Recommended questions to use during the allergy interview relate to characterization of the reaction, likelihood of causality, presence of IgE-mediated reaction, timeframe of the reaction, receipt of other antibiotics and history of skin testing. Following appropriate allergy inquiry, the patient and caregivers should be educated and the medical record updated in order to avoid further discrepancies in the future.

The studies evaluated in this review further support that many patients with previously reported beta-lactam allergies in whom allergy interview is conducted can be given a beta-lactam with no adverse events noted following administration and overall stewardship of antibiotics is positively impacted. Furthermore, cost savings have been demonstrated with no significant difference in clinical outcomes when switching from alternative antibiotics post allergy interview. Interview-based allergy clarification alone has a number of strengths and weaknesses, but has proved to be a successful method for de-labelling a significant proportion of patients (11–80%) and avoiding the need for drug challenges and skin testing [29,40,60]. In addition, for higher risk patients who require further work-up, a detailed allergy history acts as the initial intervention in a multi-step process.

## 4. Conclusions

Interview-based allergy clarifications can be used as an effective intervention in a stepwise process to potentially de-label patients with erroneously reported antibiotic allergies. These interviews can be conducted by a number of health care team members, following proper education and training. Future research should be conducted to validate standardized assessment tools in order for health care professionals to more uniformly characterize and document drug allergies. 
